# A prospective study on physical performance of Chinese chronic obstructive pulmonary disease males with type 2 diabetes

**DOI:** 10.1097/MD.0000000000027126

**Published:** 2021-09-03

**Authors:** Junhong Liu, Xiao Song, Shuangshuang Zheng, Hui Ding, Honggang Wang, Xicai Sun, Xiangfeng Ren

**Affiliations:** The Third Department of Health Care, Weifang People's Hospital, Shandong Province, China.

**Keywords:** COPD, muscle endurance, muscle strength, physical performance, type 2 diabetes

## Abstract

Currently no research is available on muscle and functional performance of chronic obstructive pulmonary disease (COPD) patients with type 2 diabetes (T2DM) in China, even though both diseases have been reported to damage motor function.

This single-center prospective study involves 55 males with COPD and T2DM and 46 males with COPD. Lung function, muscle strength and endurance of the upper limbs, and quadriceps strength of both legs were assessed using instruments. The 6-min walk (6MW) test was performed to evaluate physical performance.

Between the two groups, respiratory function of COPD patients with T2DM was worse than in those without (*P* < .05). Mean handgrip strength and muscle endurance of upper limbs and mean quadriceps strength at both 60°/s and 120°/s in COPD males with T2DM was also significantly less (*P* < .05). Mean 6MW distances of COPD patients with T2DM were significantly worse (*P* < .05), and mean pulse rate (PR) increments of COPD patients with T2DM in 6MW test were significantly higher (*P* < .05).

The combination of COPD and T2DM not only brings one more chronic disease to elderly patients but also significantly affects muscle strength and endurance as well as physical performance. Accordingly, in the management of chronic diseases, we recommend that clinicians as well as patients themselves actively control blood sugar and review them regularly with a view to reducing adverse effects on physical performance.

## 1 Introduction

COPD is primarily a disease of the respiratory system and is diagnosed based on abnormal lung function evaluated by spirometry (low forced expiratory volume) and symptoms such as dyspnea, chronic cough, and/or sputum production.^[1]^ As of 2010, approximately 329 million people (4.8% of the population) were affected with COPD globally.^[2]^ Based on epidemiological observations, COPD is more common in older people, often accompanied by multiple system chronic diseases such as T2DM.^[3]^ Patients with COPD have a higher prevalence of diabetes mellitus, at 18.7% versus 10.5% in the general population.^[4]^ COPD is characterized by persistent lung inflammation, which some have suggested spills into the systemic circulation.^[5]^ Experimental evidence has shown that lung inflammation attenuates insulin-induced suppression of hepatic glucose production and moderately impairs insulin action in peripheral tissues.^[6]^ Long-term systemic inflammation may be the cause of secondary T2DM in COPD patients. Although it is not very clear why patients with COPD are affected with T2DM more often than controls are, this coexistence makes such patients’ condition and treatment plan more complicated and often leads to some disease phenotypes that cannot be explained by a single factor. COPD often leads to a reduction in physical activity, in part due to shortness of breath and muscle wasting.^[7]^ At the same time, low levels of physical activity are associated with worse outcomes of COPD patients.^[8]^

Previous studies of the physical performance of elderly patients have often focused on a single disease at the time of enrolment. In clinical practice, we found that COPD patients with other chronic diseases such as T2DM tend to have worse muscle strength, endurance, and athletic performance. We hope to implement a controlled study to confirm whether T2DM will have an additional effect on COPD patients in terms of physical performance. This is the first systematic study on the muscle and functional performance of COPD patients with T2DM in China. We compared the muscle strength and endurance of the upper and lower limbs and the performance of the 6MW test between the two groups of patients (COPD males with T2DM and COPD-only males) to evaluate T2DM's effects on physical performance. Overall, COPD males with T2DM had worse physical performance, especially as reflected in walking distance and PR increment in the 6MW test. In the grip strength test, more COPD patients with T2DM reported wrist pain. The indicators used in this study are basic indicators of the physical strength and activity ability of the elderly. Notably, combination of COPD with T2DM not only brings one more chronic disease to elderly patients but also significantly affects quality of life. Accordingly, in the management of chronic diseases, we suggest that clinicians and patients actively control blood sugar and review them regularly to reduce the adverse effects on physical performance.

## 2 Materials and methods

### 2.1 Case collection

**Figure 1:**
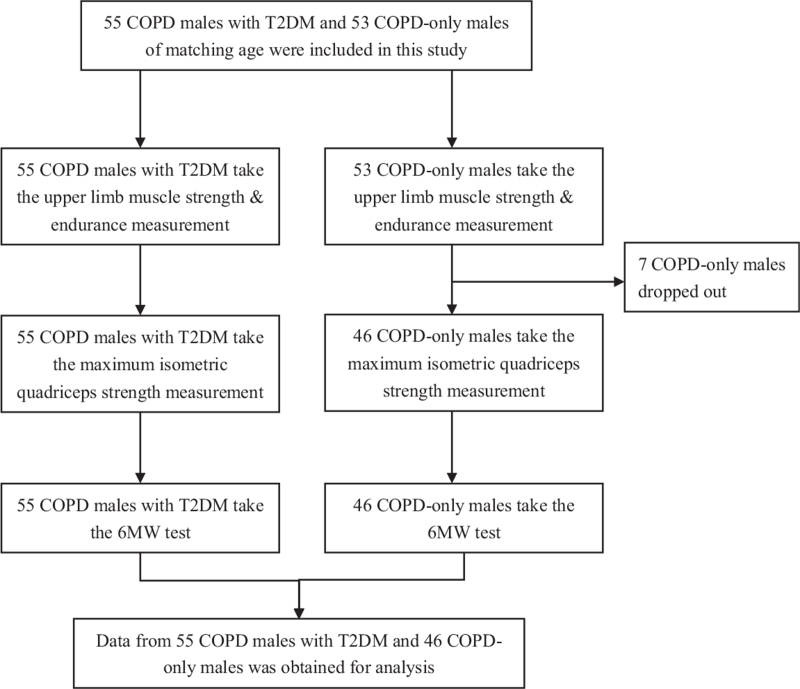
Flowchart showing the research process.

From March 2017 to October 2019, 55 COPD males with T2DM and 46 COPD-only males of matching age in Weifang People's Hospital were included in this study. The research process is shown in Figure 1. Considering the influence of gender on sports performance, we recruited only male patients with COPD. The sample size was generally calculated by GPower 3.1. Power was set to 0.8, alpha value to 0.05, and effect size to 0.54, as determined by a previous pilot study. Because the subjects were all males and met the inclusion criteria, the two groups’ allocation ratio was 1:1. It was expected that there would be at least 44 patients in each group. And we plan to recruit 1.2 times (53) of the expected sample size. However, because 7 COPD-only males could not complete all the tests, for personal reasons, this group had only 46 members. Diagnostic criteria for COPD were forced expiratory volume of 1 s, forced vital capacity (FEY 1:FVC) ratio of less than 50%, and response to bronchodilators less than 15%. The T2DM diagnostic criteria were consistent with World Health Organization criteria from 1999. Subjects with fasting plasma glucose (FPG) levels of 5.6mmol/L underwent a 75g oral glucose tolerance test.^[9]^ Patients were in clinically stable condition and had no congestive heart failure and no respiratory infection within the past 4 weeks. Patients with contraindications of Cybex, such as hypertension, cancer, or surgical recovery period, were also excluded. Patients in the COPD group were treated with first-line treatments and no extra rehabilitation, whereas those in the COPD with T2DM group were treated with first-line treatments of COPD and no extra rehabilitation. To control their T2DM, patients were taking hypoglycemic drugs, sometimes in combination with insulin injections depending on their glucose control situation. Spirometry was performed using the turbine flow-sensor–based MIR Spirolab II (Italy) by trained personnel in a quiet room, as per the guidelines of the American Thoracic Society and European Respiratory Society (ATS/ERS). All spirometries were performed at 1230–1330 h to avoid diurnal variations. The parameters measured were forced vital capacity (FVC) in liters, forced expiratory volume in 1 sec (FEV1), forced expiratory flow during 25–75% of FVC (FEF25-75%), and FEV1% predicted value (FEV1/P(%). The studies were approved by the ethics committee of Weifang People's Hospital. Written informed consent for medical record review was obtained from all patients, in accordance with the Declaration of Helsinki. To control the measurement bias caused by human errors, each of the latter measurements was completed by the same two-person research team.

### 2.2 Upper limb muscle strength & endurance measurement

A handgrip dynamometer (EH102, SENSSUN, CN) was used to measure muscle strength and the endurance of upper limbs using the previously describe technique.^[10,11]^ After having the procedure explained to them, patients were asked to hold the handgrip dynamometer in the dominant hand in a sitting position. The forearm was extended over a table and the elbow flexed at 90°. Patients were asked to hold the dynamometer so that the second phalanx was against the inner stirrup and were then asked to grip the dynamometer handle with as much force as they could. The handgrip muscle strength was recorded in kilograms, as indicated by the pointer on the dynamometer. Three recordings were taken, with a gap of 2 min between each effort, and the maximum value recorded for analysis. After 10 min rest, handgrip endurance was measured by asking the subjects to maintain their grip on the handgrip dynamometer at one-third scale as long as they could. The length in seconds for which they could maintain the grip strength was noted. Two recordings were obtained, with a gap of 5 min between each effort, and the maximum value recorded for analysis.

### 2.3 Maximum isometric quadriceps strength measurement

Lower skeletal muscle strength was evaluated using computerized dynamometers (e.g., Biodex, Cybex, Kin-Com), the gold standard.^[12]^ The quadriceps was the most common muscle group tested using this method in people with COPD.^[13–15]^ To get deeper results of lower limb skeletal muscle strength, quadriceps strength of both legs was assessed at 60°/s and 120°/s using a Cybex II dynamometer (Lumex, Ronkonkoma, NY). A standardized warm-up process was performed during a 5-minute period before Cybex testing, per the manufacturer's recommendation. At testing, the patient was sitting with a chairback angle of 85° and with knee flexion at 90°. The pelvis and thigh were secured with straps to ensure the right force mode. The leg pad was centered two fingerbreadths above the medial malleolus, with the center of rotation at the lateral joint line of the knee. The range of motion was from 0° of extension to 100° of flexion. A total of five repetitions were performed for practice before taking the definitive measurements at 60°/s and 120°/s. Between each action was a 5-min rest period. Peak torque in newton-meters (N m) was measured, with the highest value of the five measurements taken. The results showed a combination of two legs.

### 2.4 Physical performance of activities measurement

The 6MW test was performed indoors, along a flat, straight, 50-m walking course supervised by a well-trained researcher, per the ATS guidelines.^[16,17]^ Patients were encouraged during the test using the phrases “You are doing well” or “Keep up the good work ”. Patients could stop and rest during the test but were instructed to resume walking as soon as they were able to do so. If patients reported heart discomfort, dizziness, cramps, or the like, the researcher's eyes will immediately terminate the test. Oxygen saturation (SpO2) and pulse rate were assessed at the beginning and end of the test using a finger oxymeter (Yuwell, YX303, China), without supplemental oxygen. Patients were asked to grade their shortness of breath and level of fatigue using modified Borg scales^[18]^ specific to each at the beginning and end of the test.

### 2.5 Statistical analysis

Quantitative data were presented as means with standard deviation; categorical data were presented as number with percentages of the population in groups. When comparing quantitative data between groups, the Shapir–Wilk test was used to test whether the data conformed to the normal distribution. The independent sample *t*-test was used to compare the means of normally distributed quantitative data and the Mann–Whitney test to compare the means of nonnormally distributed quantitative data. The chi-squared test was used to compare the distribution of two groups of categorical variables. A *P* value <.05 was considered statistically significant. Data were analyzed using the IBM SPSS Statistics suite, version 22.0.

## 3 Results

### 3.1 Clinical characters summary

The study population included total 46 COPD-only patients and 55 COPD patients with T2DM. Table 1 gives basic information and respiratory function results for the enroled patients. We investigated three common factors potentially related to the severity of COPD. We found no significant differences between mean age, BMI, and smoking history for the two groups of COPD patients.^[18]^ Because lung function at the baseline value may affect the results of subsequent muscle strength, endurance, and 6WM tests, we compared the results of FEV1(L), FEV1/FVC (%), and FEV1/P (%) in the two groups of patients (Table 1). In general, the means of respiratory parameters in the COPD patients with T2DM were little worse than those in the COPD patients without T2DM. In particular, the mean FEV1/FVC (%) of COPD patients with T2DM was 59.8 ± 5.3 l, significantly lower than the mean value of the COPD-only group (64.7 ± 6.5, *P* < .001).

**Table 1:** Clinical Characters of COPD only and COPD with T2D patients.

Parameter	COPD only	COPD wih T2DM	*P* value
Age (yr)	69.2 ± 6.4	70.5 ± 6.7	.340
BMI (kg/m^2^)	25.9 ± 2.5	26.2 ± 3.0	.512
Smoke history(n, %)	24 (52.2%)	38 (56.4%)	.748
FEV1 (L)	1.6 ± 0.2	1.7 ± 60.2	.012
FEV1/FVC (%)	64.7 ± 6.5	59.8 ± 5.3	<.001
FEV1/P (%)	52.4 ± 7.4	49.8 ± 6.9	<.001

BMI = body mass index, FEV1/P(%) = FEV1% predicted value, FEV1 = forced expiratory volume in 1 s, FVC = forced vital capacity in liters.

### 3.2 Upper limb muscle strength & endurance and quadriceps strength results

**Figure 2:**
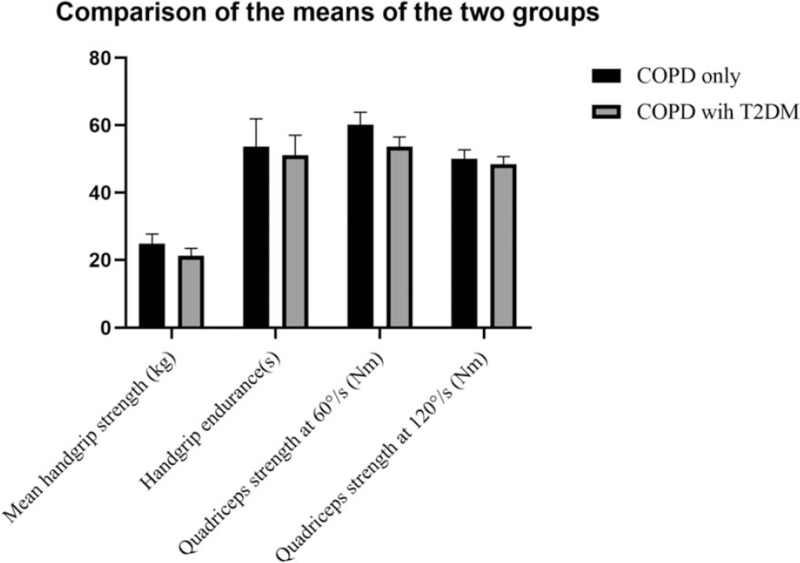
Upper limb muscle strength & endurance and quadriceps strength comparisons The handgrip muscle strength was recorded in kilograms. And the handgrip endurance was measured by asking the subjects to maintain their grip on the handgrip dynamometer at one-third of their maximum handgrip strength for as long as they could, witch unit is second. The peak torque in newton-meters (N m) was measured at at 60°/s and 120°/s to represent the quadriceps strength. And the results showed a combination of two legs. All results are the highest of the measured values.

Muscle strength and endurance are important to COPD patients’ mobility and quality of life. We estimated the upper-extremity muscles by measuring grip strength and the lower-extremity skeletal muscles by measuring the quadriceps, as has been commonly done in other studies.^[19,20]^ As Figure 2 showed, the mean handgrip strength in COPD patients with T2DM was 21.2 ± 2.2 kg and was significantly less than in the COPD-only males (24.9 ± 2.8, *P* < .001). Mean muscle endurance in COPD patients with T2DM was 51.1 ± 5.9 sec, significantly less than in COPD-only subjects (53.6 ± 8.3 sec, *P* =  < .001). It is worth noting that 3 of 55 (5.5%) COPD patients with T2DM reported mild wrist pain when using the grip. Mean quadriceps strength at 60°/s was 53.6 ± 2.9 Nm in COPD patients with T2DM, significantly less than in COPD-only patients (60.2 ± 3.7 Nm, *P* < .001). Mean quadriceps strength at 120°/s was 48.4 ± 2.3 in COPD males with T2DM, versus 50.1 ± 2.6 Nm in COPD-only males. The difference between the two was also statistically significant (*P* = .001 < .05). Through a simple test with a handheld dynamometer and planned Cybex II dynamometer test, we found that upper limb muscle strength and endurance, as well as quadriceps strength, of COPD males with T2DM were weaker than in those with COPD only. This may indicate that T2DM has a negative effect on the main muscles, but the specific path leading to such a result remains unclear. Perhaps it reflects the long-term adverse effects of T2DM itself on the muscles, or perhaps T2DM aggravates the respiratory function of COPD patients, thereby affecting the muscles’ vitality.

### 3.3 6-min walk test results

The 6MW test is a simple and widely recognized functional performance evaluation method. Table 2 shows the results of the 6-min walk test in COPD-only males and COPD males with T2DM. The mean 6MWD is 356.3 ± 38.7 m in COPD patients with T2DM, significantly less than in COPD-only patients (380.8 ± 34.7m, *P* = .001 < .05). No significant difference in SpO2(%) reduction, based on finger oximetry, or Brog scale increment were seen between two groups, which were the calculation results before and after exercise. However, the mean PR increment was 34.7 ± 9.4 beats/min in COPD patients with T2DM, significantly higher than in COPD-only patients (30.5 ± 9.2 beats/min, *P* = .028 < .05). The results of the 6MW test indicate that T2DM may adversely affect the basic physical activities of elderly males with COPD, as also reflected in the changes in center rate during the test.

**Table 2:** Detailed results of the 6-min walk test between two groups.

Parameter	COPD only	COPD wih T2DM	*P* value
6MWD(m)	380.8 ± 34.7	356.3 ± 38.7	.001
SpO2 (%) before test	96.1 ± 1.1	95.5 ± 0.9	.004
SpO2 (%) after test	93.6 ± 1.5	92.9 ± 1.3	.032
SpO2 (%) reduction	2.5 ± 1.9	2.6 ± 1.6	.903
PR(beats/min)before test	79.5 ± 7.0	77.5 ± 5.7	.123
PR(beats/min)after test	110.1 ± 6.7	112.2 ± 6.5	.101
PR(beats/min)increment	30.5 ± 9.2	34.7 ± 9.4	.028
Brog scale before test	4.2 ± 1.3	4.1 ± 1.3	.688
Brog scale after test	5.6 ± 0.9	5.7 ± 0.8	.475
Brog scale increment	1.4 ± 1.3	1.7 ± 1.3	.400

6MWD = 6-min walk distance, PR = Pulse Rate, SpO2 = Blood oxygen saturation levels.

## 4 Discussion

In 2017, 425 million people worldwide had diabetes, with type 2 accounting for about 90% of cases.^[21]^ Type 2 diabetes is characterized by insulin resistance, which may be treated with medications such as insulin sensitizers with or without insulin. Skeletal muscle is the major site of glucose disposal in response to insulin and, correspondingly, is the major site of insulin resistance in T2DM.^[22]^ Evidence suggests that mitochondrial respiration is decreased in skeletal muscle of patients with T2DM, which can lead to further decreases in skeletal muscle function.^[23]^ Also, because of the complications associated with type 2 diabetes, older people with T2DM may have more serious functional impairment than their peers, perhaps reflecting a link between the metabolic and mechanical functions of muscle.^[24]^ From the perspective of physiology and metabolism, T2DM can cause potential adverse effects on human muscles and motor functions. People with T2DM may perform worse in physical activities than people of the same age who are not suffering from the disease. Previous introduction has reviewed decreased muscle strength, decreased endurance, and poor motor function in COPD patients.^[7,8]^ From a single-disease perspective, COPD and T2DM both negatively affect the body's muscles and mobility in terms of mechanism and clinical results. However, previous studies have not asked whether the combination of the two diseases will further deteriorate motor function, especially in elderly patients. In this study, we tested whether respiratory function, muscle strength and endurance, and physical performance worsened when COPD patients also had T2DM.

We selected two groups of COPD patients whose age, BMI, and smoking history were not significantly different. Comparison of results between groups showed that respiratory function in the COPD patients with T2DM was little worse than in COPD patients without T2DM, consistent with earlier findings.^[25]^ Mean handgrip strength and endurance in COPD patients with T2DM were significantly less than in COPD-only males. Mean quadriceps strengths at 60°/s and at 120°/s in COPD patients with T2DM were also significantly less than in COPD-only patients. Muscle loss is an age- and disease-related process. Systemic diseases such as diabetes, obesity, and respiratory disorders can degrade muscle function, although the mechanism behind this degradation has not been studied clearly.^[26]^ It has been reported that blunting of coupled and uncoupled respiration in T2DM patients can be attributed to lower levels of mitochondrial content.^[27]^ Mitochondria play a central role in fuel use and energy production, and a reduction in their levels affects systemic metabolic homeostasis and muscle energy. The inflammation caused by T2DM can also play a negative role in muscle atrophy.^[28]^ T2DM may thus reduce the muscle strength and endurance of COPD patients through various mechanisms, such as metabolism, inflammation, and muscle mass reduction.

The direct effect of loss of muscle strength and endurance is deterioration of physical activity. Accordingly, we used the 6MW test to explore changes in physical activity between the two groups of patients. The 6MW test is a reliable tool for measuring the physical performance and disease severity of COPD patients.^[29,30]^ In large studies, it has been shown that walk distance is an independent prognostic factor for COPD disease.^[31]^ In our study, the 6MWD of COPD patients with T2DM was significantly less than for COPD-only patients. The mean PR increment during the 6MW test of COPD patients with T2DM was also significantly higher than for COPD-only patients. When combining the subjective report of the oximeter and the patient's dyspnea, the 6MW test result showed decreased exercise capacity and intense heart rate fluctuations after exercise in COPD males with T2DM. COPD exacerbations and mortality were associated with a low level of physical activity, which was associated with other outcomes of COPD, such as dyspnoea, exercise capacity, and FEV1.^[32]^ Accordingly, clinicians and patients should attend closely to T2DM's effects on the physical activity of COPD patients, actively controlling blood sugar to reduce the possibility of adverse outcomes.

Previous research into COPD has generally been limited to a single disease and specific outcome, with little understood about the potential harm to patients’ physical function caused by the combination of two diseases. This study shows that the combination of T2DM is not just another chronic disease but also adversely affects respiratory function and exercise outcome for COPD patients. Through auxiliary instrument tests and 6WM tests, we can objectively measure and compare differences in muscle strength, endurance, and physical activity between COPD patients with T2DM and COPD-only patients. However, this study still has some limitations, being a single-center study that did not include a sufficiently large population. We intend to observe a wider range of populations in future studies, such as young COPD patients and female patients with T2DM. By including a wider population, we can determine whether our conclusions are universal. Funding constraints prevented this study from including any intervention measures, such as planned sports training. In the future, we hope to study whether exercise training can benefit COPD patients with T2DM and COPD-only patients.

## 5 Conclusions

This is the first study to have investigated the effect of type 2 diabetes on muscle strength and endurance and on physical performance in Chinese COPD males. We compared the muscle strength and endurance of the upper and lower limbs and performance on the 6MW test between two groups of patients (COPD males with T2DM and COPD-only males) to evaluate T2DM's effects on physical performance. Overall, COPD males with T2DM showed poor performance in upper limb muscle strength and endurance and in quadriceps strength. In the grip strength test, more COPD patients with T2DM reported wrist pain. COPD males with T2DM also had worse physical performance, especially on walking distance and PR increment in the 6MW test. The indicators used in this study are basic indicators that reflect physical strength and activity ability in the elderly. The combination of COPD with T2DM not only brings one more chronic disease to elderly patients but also significantly affects their quality of life. Accordingly, in the management of chronic diseases, we recommend that clinicians and patients actively control blood sugar and review them regularly to reduce the adverse effects on physical performance.

## Acknowledgments

We appreciate the patients, the doctors and nurses who contributed to this research work.

## Author contributions

**Data curation:** Xiao Song, Honggang Wang.

**Formal analysis:** Junhong Liu, Hui Ding, Honggang Wang.

**Investigation:** Hui Ding.

**Methodology:** Xiao Song, Shuangshuang Zheng, Xicai Sun.

**Supervision:** Xiangfeng Ren.

**Validation:** Xiangfeng Ren.

**Writing – original draft:** Junhong Liu.

**Writing – review & editing:** Junhong Liu, Xiangfeng Ren.
